# Artificial pancreas: the past and the future

**DOI:** 10.1007/s10047-025-01510-1

**Published:** 2025-05-25

**Authors:** Hiroyuki Kitagawa, Masaya Munekage, Satoru Seo, Kazuhiro Hanazaki

**Affiliations:** 1 Department of Surgery, Kochi Medical School, Kohasu, Okocho, Nankoku, Kochi 783-8505 Japan; 2 Kochi Medical School Hospital, Kohasu, Okocho, Nankoku, Kochi 7838505 Japan

**Keywords:** Artificial pancreas, Sensor augmented pump, Closed loop, Automated insulin delivery, Perioperative and critically ill patients

## Abstract

In glucose management using continuous insulin infusion, artificial pancreas systems prevent blood glucose fluctuations and severe hypoglycemia using insulin pumps and continuous glucose monitoring. Advances in both insulin pumps and continuous glucose monitoring have enabled the transition from sensor augmented pump therapy, where insulin delivery is manually adjusted, to hybrid closed-loop insulin pump therapy, which automatically adjusts basal insulin infusion. Furthermore, fully automated insulin delivery systems that adjust insulin based on variations due to meals and exercise are now possible. These systems have been primarily applied to patients with type 1 diabetes but are now expanding to all insulin-dependent patients. Wearable artificial pancreas systems measure glucose levels in subcutaneous tissue fluid, while bedside artificial pancreas systems measure glucose levels in venous blood, making them suitable for managing the highly variable blood glucose levels of perioperative and critically ill patients. Future developments are anticipated to integrate the benefits of both wearable and bedside systems.

## Introduction

Patients with diabetes may experience severe hyperglycemia during perioperative periods or sick days, necessitating insulin treatment. Traditional continuous insulin infusion for strict blood glucose management poses risks of hypoglycemia due to over-administration and imposes labor burdens on medical staff, owing to the need for frequent blood sampling [1]. In contrast, artificial pancreas therapy can continuously measure blood glucose and adjust insulin doses, providing stable blood glucose management, reducing hypoglycemia risk, and alleviating medical staff workload [2].

Artificial pancreas systems come in two types: wearable and bedside. Wearable artificial pancreases reduce nocturnal hypoglycemia risk in patients with type 1 diabetes (T1D), whereas bedside artificial pancreases focus on tight glycemic control in perioperative and critically ill patients, avoiding hypoglycemia and reducing blood glucose fluctuations [3]. The artificial pancreas substitutes the endocrine function of the pancreas and is a term used in the field of automated insulin delivery (AID). This initially applied in T1D, its use has expanded to all insulin-dependent patients, including type 2 diabetes (T2D) or perioperative and severe conditions, with growing data supporting their use in hospital setting.

The development of AID systems has been driven by progress in real-time continuous glucose monitors (CGMs) and algorithms that guide and continuously adjust the insulin administration based on CGM readings and trends. The first step towards closed loop system was the development of insulin pumps with a predictive low glucose management feature, which suspends insulin delivery when low glucose levels are predicted. With further progress in CGM accuracy and pump algorithms, we have now entered the era of closed-loop insulin pumps.

This narrative review, titled “Artificial Pancreas: The Past and the Future,” aimed to explore the functions of reported artificial pancreases, evidence supporting their role in blood glucose management in patients with diabetes requiring insulin administration and perioperative and severe patients, as well as recent research and prospects in the field of artificial pancreases.

## Advancements in insulin pumps and CGMs in wearable artificial pancreas systems

Wearable artificial pancreases consist of insulin pumps that perform continuous subcutaneous insulin infusion (CSII) stably and CGMs that continuously measure glucose levels. This involves the use of a small insulin pump and infusion circuit attached subcutaneously to the abdomen or buttocks, allowing for adjustable basal insulin delivery. CSII reduces patient pain and effort, because the pump only needs to be attached to a needle once every 2–3 days and does not need to be removed during sports or when bathing. Recently, some pumps that can be replaced as often as once every 7 days has been introduced. In addition, during strenuous contact sports or bathing, the pump must be removed, but the needle does not, so pen insulin injections can be substituted in the meantime. Some models are designed to reduce the psychological burden on the user, such as a patch-type pump that can be worn on the abdomen and operated with a smartphone-type remote control (Table 1).

**Table 1:** Representative wearable artificial pancreas and their features

Device, Manufacturer	Pump	CGM	Features
MiniMed^™^ 780G Medtronic	MiniMed™ 780G insulin pump	Guardian^**™**^ 4	The system automatically adjusts the amount of basal insulin every 5 min in response to the glucose value measured by the sensor, and automatically injects corrective insulin. Infusion set can be used for up to 7 days (https://www.medtronicdiabetes.com/products/minimed-780g-insulin-pump-system)
Control-IQ Tandem Diabetes Care	t:slim X2	Dexcom G6/G7 FreeStyle Libre 2 Plus	Based on CGM readings, glucose levels can be predicted 30 min ahead and automatically corrected for basal insulin secretion and bolus dosing. The battery life is up to 7 days (https://www.tandemdiabetes.com/products)
Omnipod 5 Insulet Corporation	Pod	Dexcom G6/G7 FreeStyle Libre 2 Plus	Tubeless and waterproof insulin pump allows insulin administration for up to 72 h. It can be linked to a smartphone to monitor and control glucose levels and insulin dosing. (https://www.omnipod.com/what-is-omnipod/omnipod-5)
DBLG1 Diabeloop	Dana-i	Optimal devices	There are dedicated smartphone-type devices equipped with algorithms that control insulin dosage based on 5-min glucose values obtained from the CGM, the patient's condition, blood glucose trends, and dietary and activity data (https://www.dbl-diabetes.com/dblg1system-dana-i)
CamAPS FX CamDiab	mylife YpsoPump	Dexcom G6 FreeStyle 3	Algorithm predicts insulin requirements for the next 2.5–4 h and adjusts insulin dosing every 8–12 min. (https://camdiab.com/faq)

Insulin pumps can be classified based on their form factor into tethered and patch types. In addition, there are single-hormone pumps that deliver only insulin and dual-hormone pumps that can deliver both insulin and glucagon. A tethered insulin pump continuously delivers insulin through a tube. The pump, about the size of a mobile phone, is worn under clothing. It administers basal insulin and bolus insulin delivery at mealtimes or during periods of high blood glucose with the push of a button. The insulin dosage and timing can be easily adjusted to fit one's lifestyle, including meals and exercise. Some models are integrated with CGMs, which measure blood glucose levels in real-time and automatically adjust insulin delivery. However, there is a risk of the tube becoming tangled or detached. In contrast, patch pumps are directly attached to the body, do not require a tube, and operated via a remote control. The advantages include a discreet appearance, easy application, and minimal interference with daily activities. However, it is important to regularly change the pump's location and monitor for any skin issues [4].

A single-hormone pump is a device that administers only insulin. It has a simple structure and is easy to operate; however, managing blood glucose levels can be challenging with insulin-only delivery [5]. In contrast, dual-hormone pumps that can deliver both insulin and glucagon help prevent rapid fluctuations in blood glucose levels and enable more stable glucose management. However, these systems are complex and require advanced skills for operation and management [6].

CGM involves wearing a sensor that measures glucose concentration on the arm or abdomen to continuously monitor glucose levels. It does not directly draw blood to measure blood glucose levels but measures glucose concentration from the interstitial fluid in the subcutaneous tissue to pseudo-predict blood glucose levels. The time lag between blood glucose levels and the glucose concentration in the interstitial fluid is about 5–20 min, but if the blood glucose level changes rapidly, the glucose concentration in the interstitial fluid may not reflect the same degree of change [7].

CGM accuracy can be affected by certain conditions which alters blood flow and interstitial fluid dynamics including severe edema, inflammation or infection (increased vascular permeability), critical illness, dehydration, and peripheral vascular disease [8].

Compared with conventional self-monitoring of blood glucose, it not only reduces the burden on the patient [9] but also improves the time rate of achieving the target blood glucose range calculated from the continuous blood glucose levels measured with CGM (time in range; TIR). Studies have reported that TIR correlates with HbA1c levels [10]. In addition, the estimated HbA1c value calculated from the mean glucose value obtained via CGM serves as a glucose management indicator (GMI), providing a shorter-term measure of blood glucose control than HbA1c [11].

With CGM, it is now possible to obtain not only TIR but also other indicators, such as the percentage of time above the target blood glucose range (time above range; TAR) and that below the target blood glucose range (time below range; TBR). In other words, a higher TIR with lower TAR and TBR indicate a safer and higher quality glycemic control. Furthermore, studies have reported that lower TIR correlates with diabetic and cardiovascular complications and prognosis [12, 13]. Recently, the index “time in tight range” and percentage of TIR (70–140 mg/dL) have also been reported as a goal for higher quality glycemic control [14]. Conversely, rather than uniformly targeting a high TIR, goals should be set based on disease status, such as a TIR of at least 50% and TBR < 1% for 70–180 mg/dL in the elderly and patients with high-risk diabetes [15]. A new comprehensive measure of the risk of hypoglycemia and hyperglycemia based on CGM data, the glycemia risk index (GRI) has been reported. The GRI was calculated using the hypoglycemic components of very low (< 54 mg/dL) and low (54–70 mg/dL) glycemia, and the hyperglycemic components of very high (> 250 mg/dL) and high (180–250 mg/dL), and the formula is as follows; GRI = (3.0 × VLow) + (2.4 × Low) + (1.6 × VHigh) + (0.8 × High) [16].

CGM is of two types: professional and personal CGMs. In professional CGM, measurement results are reviewed retrospectively by both the healthcare provider and patient, whereas in personal CGM, the patient can review the data directly. In addition, personal CGMs include intermittently scanned CGM (isCG) and real-time CGM (rtCGM). rtCGM has been reported to be superior to isCGM in reducing hypoglycemia events and improving the quality of glycemic control [17]. To obtain accurate data from the CGM to calculate GMI and other parameters, 70% of the 14 days or at least 10 days of data should be obtained [18].

### Sensor augmented pump (SAP)

In SAP, the insulin pump and CGM work in tandem to provide insulin therapy; changes in blood glucose levels are predicted from glucose concentrations measured by CGM, and insulin administration is stopped if hypoglycemia is anticipated and resumed if necessary [19]. Bolus insulin administration is necessary when blood glucose levels change rapidly with meals. Recently, systems that can automatically adjust basal and bolus insulin doses by controlling the CGM and insulin pump with a computer algorithm have been developed. Even in elderly patients with T1D at high hypoglycemia risk, SAP can increase TIR and minimize TBL [20].

### Hybrid closed-loop insulin pump system (HCL)

The HCL system, in addition to the features of SAP, automatically adjusts the basal insulin delivery based on CGM data. While manual input for additional insulin is still required, overall management is automated. It increases the TIR and reduces the risk of hypoglycemia and hyperglycemia. Unlike SAP, which primarily focuses on blood glucose monitoring and alert functions with manual insulin adjustment, HCL automatically adjusts basal insulin delivery, achieving higher automation. This results in less user intervention and easier blood glucose management [21]. Wadwa et al. randomized children with T1D and compared an artificial pancreas group with a control group in which blood glucose was controlled by conventional insulin pump therapy and frequent insulin injections in a 2:1 ratio for 13 weeks of treatment at a multicenter. Results showed that the artificial pancreas group had a 12% higher TIR of 70–180 mg/dL than the control group. In particular, the TIR was 18% higher in the artificial pancreas group between 10 pm and 6 am, suggesting the advantage of the artificial pancreas in safety during the nighttime, when detection of hypoglycemia is challenging. The artificial pancreas group demonstrated better HbA1c (7.0 and 7.5%) and mean blood glucose (155 and 174 mg/dL) [22].

In addition to auto-adjustment of basal insulin, the advanced hybrid closed-loop (AHCL) system can also auto-inject corrected bolus insulin. Daly et al. conducted a two-phase crossover randomized controlled trial in 26 patients with T2D with HbA1c ≤ 12%. The patients were assigned to artificial pancreas group: switched to standard therapy with multiple daily insulin injections after 8 weeks of trial use of the artificial pancreas, and the control group that first received standard therapy then switched to the artificial pancreas after 8 weeks. Compared with the control group, the artificial pancreas group had better results in TIR (70–180 mg/dL, 66.3 vs. 32.3%; *P* < 0.001), TAR (33.2 vs. 67.0%), mean blood glucose (166 mg/dL vs. 227 mg/dL; *P* < 0.001), HbA1c (7.3 vs. 8.7%; *P* < 0.001), and comparable TBL (0.44 vs. 0.08%; *P* = 0.43) [23].

In a pragmatic observational study conducted at 31 centers in the United Kingdom, switching from isCGM to HCL insulin delivery systems reduced HbA1c by an average of 1.7% among 520 HCL users with a median follow-up of 5.1 months. The HbA1c decreased by an average of 1.7%, TIR (70–180 mg/dL) increased from 34.2 to 61.9%, and those with TBR < 4% increased from 0.8 to 28.2%, and nearly all participants reported that HCL therapy had a positive impact on their quality of life [24].

In an observational study of glycemic control in children with T1D using AHCL systems (Medtronic MiniMed^™^ 780G and Tandem Control IQ), TIR at week 4 of AHCL increased (780G: from 65.7 to 70.5%, *p* < 0.01 and Control IQ: from 64.8 to 70.1%, *p* < 0.01, respectively) [25]. The CGM data from 4120 patients using the AHCL system (MiniMed^™^ 780G) showed that the mean GMI: 6.8%, TIR: 76.2%, TBR < 70: 2.5%, TAR > 180: 21.3, 77.3 and 79.0% of users achieved TIR > 70% and GMI < 7.0%, respectively [26].

We have described a system that administers insulin alone, recently, a dual-hormonal system that administers glucagon along with insulin is expected to reduce the risk of hypoglycemia compared to conventional insulin pump therapy [27]. Compared with insulin pump therapy, the dual-hormonal AP had higher TIR, without meal or exercise announcements in adults with T1D [28]. The use of a dual-hormone artificial pancreas in patients who underwent total pancreatectomy has been reported to increase the TIR (70–180 mg/dL) compared to conventional insulin therapy (78.30 vs. 57.38%) and to decrease the incidence of hypoglycemia (0.00 vs. 1.61%; *P* = 0.004) [29]. To establish dual-hormonal artificial pancreas, it is necessary to measure the change in glucose values with exogenous glucagon administration, in addition to glucose absorption in the gastrointestinal tract and absorption of insulin administered subcutaneously [30].

## Glycemic management in perioperative and critically ill patients

Perioperative and critically ill patients have stress-induced hyperglycemia due to elevated stress hormones and increased insulin resistance. Previous studies recommended that blood glucose levels should be controlled below 150 mg/dL [31]. In thoracic and cardiac surgery, preoperative diabetes mellitus and hyperglycemia > 200 mg/dL within 48 h of surgery were risk factors for surgical site infection (SSI) [32]; moreover, preoperative blood glucose levels ≥ 126 mg/dL increased the risk of mediastinal infection after cardiac surgery [33]. Perioperative glycemic control is also important in general surgery, as preoperative HbA1c of ≥ 7.0% increased the risk of infectious complications [34], and postoperative hyperglycemia increased SSI risk and was a risk factor for prolonged hospital stay, sepsis, renal failure, and surgical mortality [35, 36]. Patients with diabetes were more prone to postoperative hyperglycemia than those without diabetes; however, some reports have shown that the incidence of postoperative infectious complications was higher in patients without diabetes [37]. Stress invasion, especially in perioperative patients undergoing esophageal and hepatobiliary surgery, as well as critically ill patients, leads to impaired glucose tolerance and insulin sensitivity due to cytokine fluctuations and catecholamine secretion [38]. In addition, perioperative treatments such as steroids and nutritional therapy are often associated with hyperglycemia [39].

Currently, no clear and uniform evidence regarding the optimal perioperative blood glucose targets to prevent SSIs has been reported; the Centers for Disease Control and Prevention recommends blood glucose control < 200 mg/dL [40]. The American College of Surgeons and Surgical Infection Society recommends that the target blood glucose range be controlled to 110–150 mg/dL (< 180 mg/dL for cardiac surgery) [41]; the American diabetes association recommended target glucose range of 110–180 mg/dL for patients with cancer and diabetes [42]. In 2001, van den Berghe et al. compared the intensive insulin therapy (IIT) group of surgical ICU patients with blood glucose levels of 80–110 mg/dL with a control group managed at 180–200 mg/dL and reported lower mortality in the IIT group [43]. However, the Leuven II study of IIT in Medical ICU patients reported a 2.7% reduction in hospital mortality, although no significant difference was observed (37.3 vs. 40.0%, *P* = 0.33) [44]. Subsequent IIT studies, such as VISEP and GLUCONTROL, revealed a risk of severe hypoglycemia [45, 46]. In the NICE–SUGAR study, both 90-day mortality (27.5 vs. 24.9%; *P* = 0.02) and severe hypoglycemia incidence below 40 mg/dL (6.8 vs. 0.5%; *P* < 0.001) were higher in the IIT group (81–108 mg/dL) compared with the control group (< 180 mg/dL) [47]. Consequently, IIT was associated with an increased risk of hypoglycemia, but with limited prognostic benefit for clinically ill patients [48]. For safety against hypoglycemia, maintaining blood glucose levels within 110–150 mg/dL is recommended. The Society of Critical Care Medicine and The Society of Thoracic Surgeons recommend initiating insulin administration when blood glucose levels reach 180 mg/dL and targeting a range of 140–180 mg/dL to prevent hypoglycemia [49, 50].

Recently, the use of CGM in glucose management for critically ill patients has been reported to significantly reduce the incidence of hypoglycemia, overall mortality, and glucose variability compared to traditional point-of-care measurements [51], and TIR and TBR have gained more attention than blood glucose. For critically ill patients without diabetes, maintaining a TIR of 70–140 mg/dL > 80% is recommended [52], while a TBR of < 1% is recommended for elderly and high-risk patients with diabetes.

Gunst et al. compared the tight glucose control (80–110 mg/dL) using the LOGIC–Insulin algorithm vs. liberal glucose control (initiating insulin only when glucose > 215 mg/dL) in 9230 critically ill patients not receiving early parenteral nutrition. In results, median morning glucose levels were 140 mg/dL in the liberal control group and 107 mg/dL in the tight control group, and severe hypoglycemia occurred in 0.7% of the liberal-control group and 1.0% of the tight-control group; however, no significant difference in ICU stay length or 90-day mortality between the two groups [53].

Tight glycemic control in critically ill patients requires careful consideration of the benefits of treatment vs. the risk of hypoglycemia. As recent advances in CGM and algorithms have enabled individualization of glycemic management, an individualized approach to glycemic management will also be important for ICU patients [54].

The importance of considering changes in blood glucose levels before admission and after ICU admission in ICU patients has been reported. If the HbA1c level before admission was less than 6.5%, the higher the blood glucose, the higher the ICU mortality rate, but the lower the blood glucose level for 8.5% or higher, the higher the mortality rate [55]. Relative hypoglycemia was defined as a decrease in blood glucose levels in the ICU by 30% or more compared to before hospitalization, or when the HbA1c level before hospitalization was 8.0% or higher and the blood glucose level in the ICU was 70–110 mg/dL and was considered a factor that increased the risk of mortality [56, 57]. In addition, the average blood glucose level during ICU admission was compared with the blood glucose level before hospitalization, and the risk of mortality was evaluated as glycemic ratio (GR). The GR had strong correlations with hyperglycemia, TIR, and mean glucose level, but moderate correlations with HbA1c [58], and there was a J-shaped relationship between GR with mortality [59]. Okazaki et al. reported that there was a J-shaped association between the maximum GR, defined as maximum blood glucose values during the first 24 h after ICU admission divided by HbA1c-derived average glucose and mortality, while U-shaped association was showed in the minimum GR [60]. In diabetic patients, persistent hyperglycemia impairs glucose sensitivity in neurons and pancreatic β cells, increasing the hypoglycemia detection threshold in the brain, and a process that leads to relative hypoglycemia has been proposed [61].

## Perioperative blood glucose control with bedside artificial pancreas

Bedside artificial pancreas (STG-55; Nikkiso Co., Ltd., Japan) performs continuous blood sampling at a minute volume of < 2 mL/h, and a glucose sensor measures blood glucose levels in real time. Using an algorithm based on blood glucose levels and change rate, glucose or insulin is injected to maintain a set target blood glucose range [62]. Basic animal studies have demonstrated the ability of bedside artificial pancreas to maintain blood glucose within the target range with errors limited to 21% compared with conventional methods [63, 64]. In clinical settings, the STG-55 system achieved a TIR exceeding 75% [65] and had a lower elevation of postoperative white blood cell counts than the control group [66] following esophagectomy. Furthermore, the STG-55 decreased the need for frequent blood sampling and subcutaneous insulin infusion, reducing the workload of nurses and the risk of wrong insulin dosing [67]. It also provided stable postoperative glycemic control with low glycemic variability and prevented ketoacidosis in patients with esophageal cancer with T1D [68].

Post-pancreatectomy blood glucose management using the STG-55 was reported to significantly reduce the incidence of severe complications compared to conventional sliding scale insulin management [69]. Intraoperative STG-55 use has also revealed that blood glucose levels increase rapidly with ischemia–reperfusion in cardiac surgery using an artificial heart–lung or liver surgery using the Pringle technique [70, 71]. In cardiac surgery, the use of an STG-55 resulted in less hypoglycemia and glycemic variability compared to patients treated with continuous intravenous infusion of insulin [72].

Glycemic management with a bedside artificial pancreas is suitable for the patients with after esophageal, liver, pancreatic, and cardiac surgeries, where stable blood glucose control might be difficult with conventional insulin continuous injections, and for patients with T1D [73].

## Challenges and future prospects of artificial pancreas

To spread artificial pancreas therapy, there are issues such as cost, training of staff who handle it, and handling of the huge amount of data recorded with consideration for personal information [74]. One major disadvantage of bedside artificial pancreas is treatment interruption owing to continuous blood sampling failure. To prevent poor blood sampling and glucose contamination in the infusion route, the continuous blood sampling catheter placement site is generally determined in a linear vein in the forearm, away from the joint and downstream of the infusion route. To resume artificial pancreas therapy, several steps are required, including the re-establishment of the blood-sampling route, which increases the workload of busy ICU nurses and disrupts the planned course of artificial pancreas therapy. To address this issue, we have provided a manual that can be shared by nurses and clinical engineers involved in operating the artificial pancreas. This manual provides guidance on the smooth preparation of the device, inter-professional transfer of the device, and response to continuous blood sampling failure [75].

Another drawback of the bedside artificial pancreas is its lack of mobility. Ideally, when patients return to the general ward from the ICU and resume rehabilitation and diet; their blood glucose could be managed with a wearable artificial pancreas. However, the glucose concentration of CGM reflects the blood glucose level 5–10 min prior, which may delay the detection of hyperglycemia or hypoglycemia [76]. Insulin pumps and CGMs should also be removed during radiography owing to the risk of malfunction from electromagnetic radiation.

In this regard, non-mechanical and protein-free devices applied to the skin have been investigated including a prototype microneedle-type artificial pancreas fused with glucose-responsive gel and regenerated silk fibroin [77], and an electronics-free, synthetic boronated gel-based insulin-diffusion-control device, in which boronic acid, which reversibly binds to glucose, is incorporated into a polymer gel [78]. Recently, the accuracy of CGM in measuring glucose levels has improved. A study evaluated the accuracy of the Dexcom G6 sensor in patients after abdominal surgery and solid organ transplantation, using arterial blood glucose levels as the reference. The results reported a mean absolute relative difference of 9.4%, a relative bias of 1.4%, a TIR of 78%, and a TBL of less than 1% [79].

Artificial pancreas has enabled safe and effective glucose management and improved the patient's quality of life through algorithmic automation and miniaturization. Further research is expected to improve the equipment and create new innovations. In conclusion, safe, high quality, and totally automatic blood glucose management by artificial pancreas is ideal; however, it requires continuous multidisciplinary medical staff cooperation and education to ensure its proper function; moreover, it is important to accommodate the patient's preferences.

## Data Availability

This article is a review and does not include any original data. Therefore, no data are available.
